# Umbilical artery aneurysm without aneuploidy and delivery of a live neonate

**DOI:** 10.1515/crpm-2021-0091

**Published:** 2022-05-27

**Authors:** Gregory K. Lewis, Josette C. Dawkins, Xiangna Tang

**Affiliations:** Department of Obstetrics and Gynecology, Rochester General Hospital Rochester NY, USA; Department of Obstetrics and Gynecology, Rochester General Hospital, Rochester NY, USA

**Keywords:** aneuploidy, cesarean delivery, ultrasound, umbilical artery aneurysm

## Abstract

**Objectives:**

Umbilical artery aneurysm, a rare structural anomaly of the umbilical cord, is frequently associated with fetal aneuploidy, fetal growth restriction and fetal demise. At present there are no definitive protocols or guidelines for the surveillance and management of this condition.

**Case presentation:**

The index case is an 18-year-old primigravida who had an ultrasound at 35 weeks and 5 days gestation due to lagging symphysio-fundal height measurement. The ultrasound scan revealed a normal fetus with estimated fetal weight that was appropriate for gestational age. There was a cystic structure with internal echoes originating from the placenta at the point of the umbilical cord insertion, which was determined to be a 1.9 × 1.8 cm umbilical artery aneurysm on 3D and Doppler imaging. On follow up imaging the aneurysm had increased in size and measured 3.06 × 1.79 cm. The patient subsequently had a cesarean section delivery of a live female. Karyotyping subsequently revealed 46 XX.

**Conclusions:**

A total of 15 cases of umbilical artery aneurysm have been reported in the literature to date, of which there were 5 live born infants with normal karyotype. The remaining 10 cases were intra-uterine fetal demise or trisomy 18 with subsequent neonatal deaths. When monitoring the aneurysms with ultrasound, change in size and Doppler indices play a pivotal role in helping to determine time and mode of delivery and thus allow for a favorable perinatal outcome.

## Introduction

Umbilical artery aneurysm is a rare structural anomaly of the umbilical cord and is frequently associated with fetal aneuploidy, fetal growth restriction and fetal demise [1]. Umbilical artery aneurysm has been found to be associated with trisomy 18 syndrome and is due to the abnormal placental vasculature seen in these pregnancies [2]. Euploid fetuses with umbilical artery aneurysms have high rates of demise occurring between 26 and 34 weeks gestation. It is quite difficult to ascertain the exact etiology and predict the timing of greatest risk of intrauterine fetal demise (IUFD) [3].

Technological advances with higher resolution images and detailed prenatal evaluations have revolutionized the diagnosis of umbilical artery aneurysm [1]. At present there are no definitive protocols or guidelines for the surveillance and management of this condition. Important clinical parameters such as the influence of size and location of the aneurysm on perinatal outcomes, appropriate timing and mode of delivery are yet to be fully elucidated [4]. Such information would certainly facilitate better patient counseling and plan for more comprehensive allow for better counseling of patients and for a more comprehensive multidisciplinary team involvement including perinatology, neonatology, and the medical genetics team. Here we report a case of umbilical artery aneurysm diagnosed in the third trimester and subsequent delivery of a live neonate with normal karyotype and will highlight aspects of management reviewed in the literature.

## Case presentation

**Figure 1:**
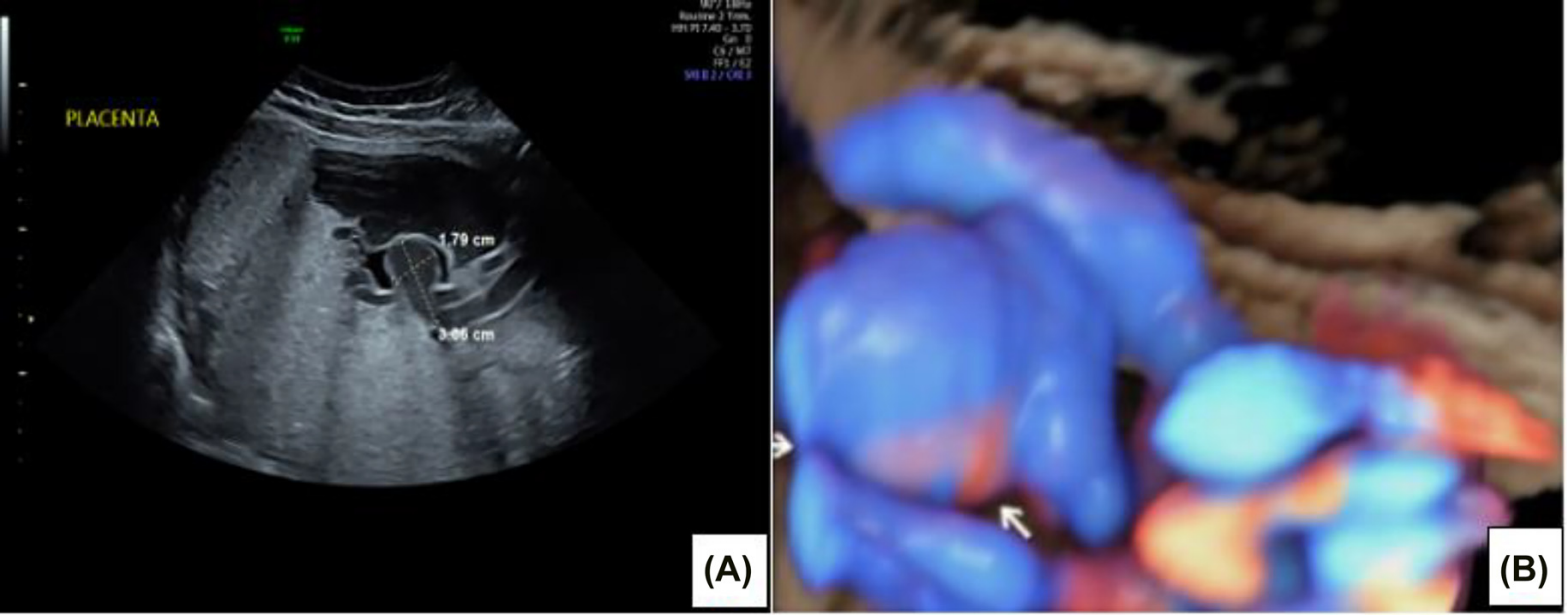
Umbilical artery aneurysm on 3D and Doppler imaging. (A) Cystic structure near cord insertion to the placenta. (B) 3D ultrasound representation of the aneurysm.

**Figure 2:**
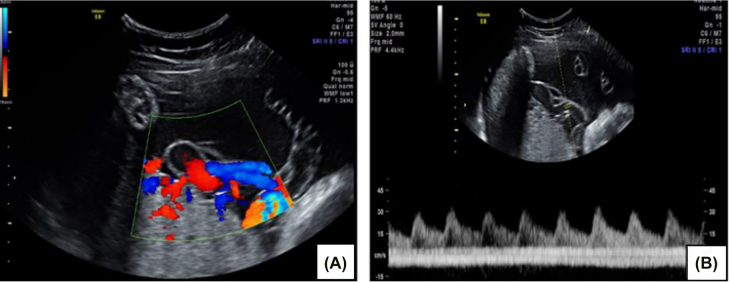
Color (A) and pulse wave (B) Doppler flow through the umbilical artery aneurysm.

The index case is that of an 18-year-old primigravida who was referred to the antenatal testing center for a growth scan at 35 weeks and 5 days gestation, due to lagging symphysio-fundal height measurement. She had no significant past medical history, and her antenatal course was uncomplicated. A detailed ultrasound scan was done and revealed a normal fetus with estimated fetal weight that was appropriate for gestational age, normal amniotic fluid index and a posterior placenta with a 3-vessel umbilical cord. There was a cystic structure with internal echoes, originating from the placenta at the point of the umbilical cord insertion, which was determined to be a 1.9 × 1.8 cm umbilical artery aneurysm on 3D and Doppler imaging (Figure 1A and B). On subsequent imaging 3 days later at 36 weeks and 1 day, the aneurysm was noted to measure 3.06 × 1.79 cm, an increase in size from prior measurement. Umbilical artery Doppler studies were within normal limits (Figure 2A and B). The middle cerebral artery peak systolic volume calculated was 1.04 MoM, which was at the higher end of the normal range. Fetal echocardiogram was done and revealed normal cardiac evaluation, with no abnormality detected in the ductus venosus velocimetry.

Due to the increase in size of the aneurysm over a short interval, the patient was counseled regarding the risks of intra-uterine fetal demise and was transferred to the labor and delivery unit. Fetal monitoring via non-stress test revealed fetal tachycardia, with a baseline heart rate above 165 bpm and regular uterine contractions. Her calculated Bishop score was 2, she was remote from delivery with a category 2 tracing, and a potentially compromised fetus. The decision was then made to proceed with cesarean section delivery. A live female infant was delivered weighing 2,850 g; Apgar scores were 7 at 1 min and 9 at 5 min. The baby was admitted to the special care nursery for observation due to transient tachypnea. The newborn hemoglobin was 15.8 g/dL. Karyotyping subsequently revealed 46 XX.

**Figure 3:**
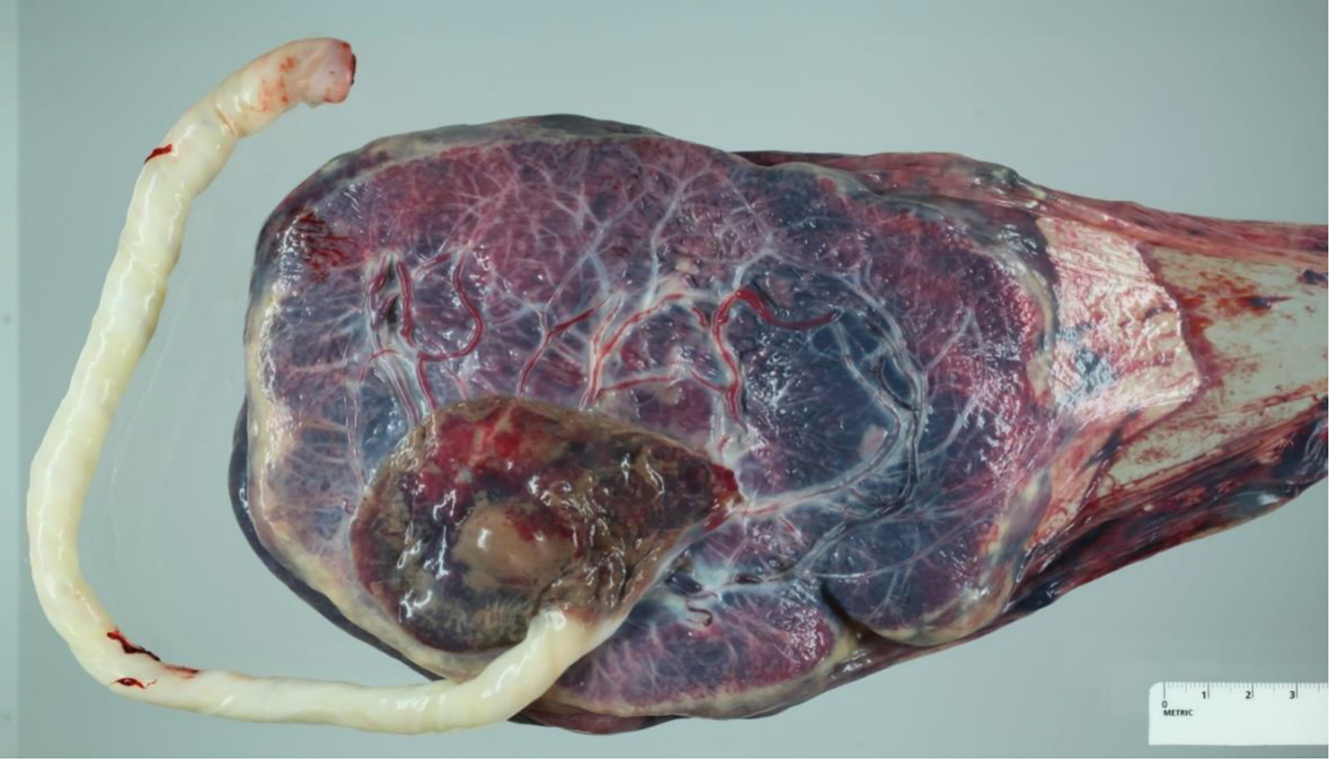
Aneurysmal area near the cord insertion measuring 2.5 × 2.4 × 1.2 cm.

**Figure 4:**
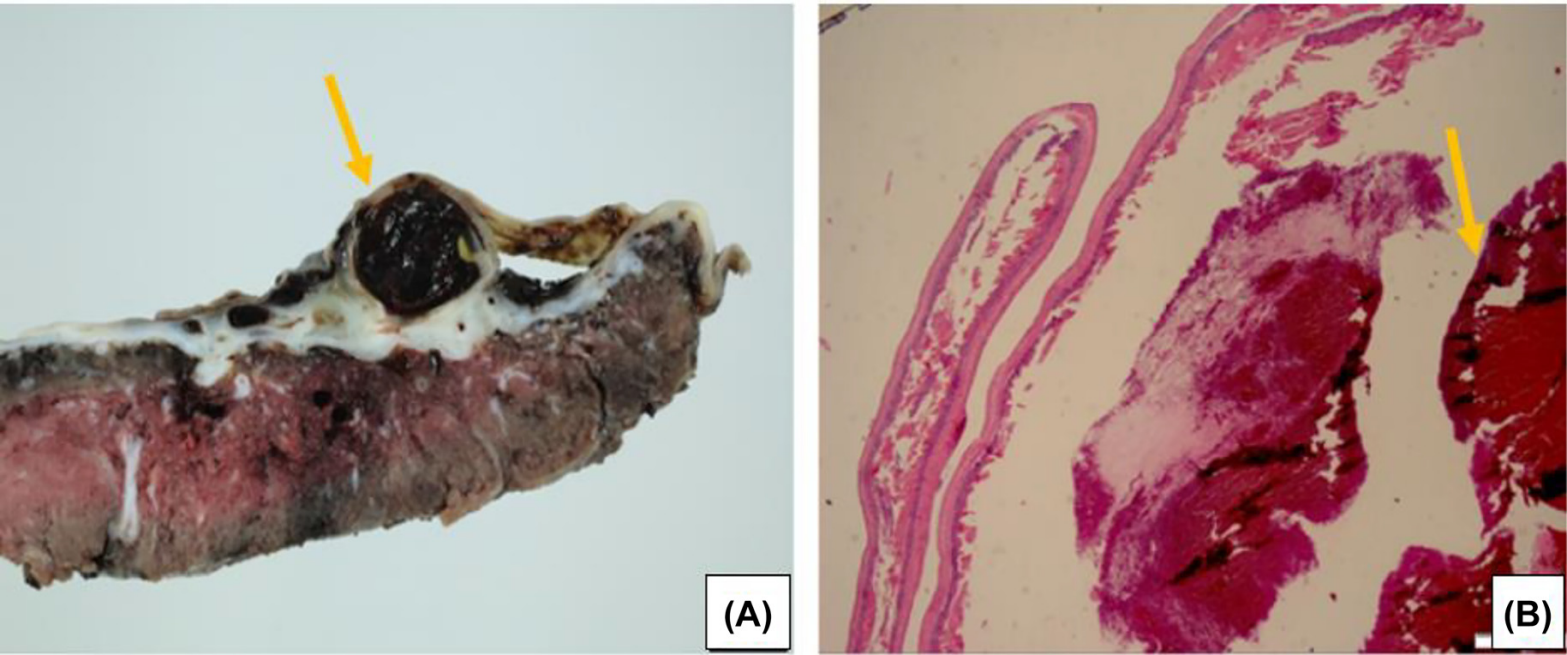
Showing thrombus within the aneurysmal vessel. (A) Umbilical artery aneurysm with a thrombus in the dilated vessel. (B) Histopathologic representation of clot within aneurysm (formed prenatally).

On pathological examination, the placenta was ovoid in shape with an eccentrically inserted 22 cm long 3-vessel edematous umbilical cord (Figure 3). There was a 2.5 × 2.4 × 1.2 cm dilated intact vessel containing a soft dark red blood clot, approximately 0.4 cm from the base of the umbilical cord (Figure 4A and B). Surrounding the blood vessel was a 7.0 × 6.0 cm ovoid pale tan area. The umbilical artery aneurysm was confirmed at the point of insertion onto the placental disc. Microscopic and macroscopic chorionic pseudocysts were also noted.

## Discussion

A total of 15 cases of umbilical artery aneurysm have been reported in the literature to date, of which there were 5 live born infants with normal karyotype [2]. Our case represents the sixth live born infant with normal karyotype. The remaining 10 cases were IUFD or trisomy 18 with subsequent neonatal deaths. From the reported cases 53% of patients with umbilical artery aneurysm had a single umbilical artery, 23% had trisomy 18 and an IUFD risk overall of 47% [2, 4]. Typically, umbilical artery aneurysms are seen at the insertion of the cord, close to the placental insertion (as was noted in this case) or close to the fetus [4]. Four other cases of umbilical artery aneurysm affecting structurally normal fetuses with a 3-vessel umbilical cord and without aneuploidy have been highlighted in the literature. The site of the aneurysm in relation to the cord was noted to be in the middle in 2 cases [5, 6]; 3 cm from the fetal end in one case [4] and at the placental site insertion in the other case [7]. In terms of overall prognosis, one fetus with the aneurysm located in the middle of the cord ended in an IUFD [5], therefore including this case, four structurally normal fetuses, without aneuploidy and a 3 vessel umbilical cord ended in live births.

It has been proposed that the poor fetal outcome associated with umbilical artery aneurysm is likely as a result of hemodynamic changes related to compression of the rest of the cord by the aneurysm, thrombosis or rupture [8]. The underlying etiology surrounding the development of an umbilical artery aneurysm is unknown but may be related to congenital thinning of the arterial wall, or a degeneration of the normally protective Wharton’s jelly, which would normally protect the vasculature and prevent the formation of an aneurysm [7]. The fetal and placental sites of cord insertion therefore represent points of weakness for aneurysmal formation. Microscopic and macroscopic pseudocysts are often seen associated with umbilical artery aneurysm and likely represents degeneration of Wharton’s jelly [2] and was also noted on pathologic evaluation of the placenta in this case.

In previously published case reports, the sizes of the reported umbilical artery aneurysms have ranged from 1.9 cm to 8 cm [9]. The impact of size may be more important in cases where only a single umbilical artery is present, as the aneurysmal vessel can potentially cause significant compression of the umbilical vein. In addition, the aneurysm may well lead to increased vascular resistance to flow in the umbilical artery and thereby increase systemic pressure, culminating in severe intra-uterine growth restriction or cardiomegaly [8].

The index patient was delivered at 36 weeks and 1 day after noting an increase in size of the aneurysm over a short interval, in conjunction with a category 2 fetal heart rate tracing. Other authors have proposed delivery as soon as fetal lung maturity is achieved, or even as early as 32 weeks if the diagnosis of umbilical artery is made early [1, 4]. Understandably such measure would likely decrease the risk of IUFD, as only three live births at 36 weeks or above have been reported including this case. Timing of delivery however still remains controversial with no uniformly generalized consensus. In keeping with current guidelines, antenatal corticosteroids administration is a reasonable consideration for patients expected to deliver prior to 37 weeks gestation and is definitely recommended for those less than 34 weeks [10]. The patient in the index case was delivered expeditiously by cesarean section prior to completion of the antenatal steroid course as she had a category 2 fetal heart rate tracing, on the background of an increase in size of the umbilical artery aneurysm.

Interestingly, in the last five reported cases, elective cesarean section was the mode of delivery chosen and whilst this seems to be the trend more data is required to make a generalized recommendation [2]. Although this patient was delivered by primary cesarean section due to the possibility of a ruptured expanding aneurysm, it is unclear whether this mode of delivery as a whole would improve fetal outcome. However, due to the higher risk of fetal demise, shared decision making should be employed, and all available data presented to the patient. The theory that an expanding aneurysm may possibly rupture when the umbilical cord comes under tension due to fetal descent during a vaginal delivery is quite reasonable and may be related to the size of the aneurysm. Two cases of aneurysmal rupture have been reported, with sizes of 6 and 9 cm, both cases ending with cesarean section deliveries. One baby had mild anemia and thrombocytopenia and had an uneventful neonatal course; whilst the other baby developed severe anemia, disseminated intravascular coagulation, and later died [7, 9]. In fact, Matsuki and colleagues went on to suggest cesarean delivery for aneurysms greater than 5 cm in diameter, further specifying that a classical cesareans section or a transverse uterine fundal incision be considered [9]. Such recommendations may be prudent due to lack of robust clinical evidence to suggest otherwise at this time. In this case the aneurysm was less than 5 cm (approximately 3 cm) and was noted to be intact on pathological evaluation.

Each case of umbilical artery aneurysm should have its management individualized to facilitate a safe outcome for both mother and baby as there is no great wealth of current evidence to guide definitive management. Such optimization should therefore include very close fetal surveillance via (1) ultrasonography, including Doppler studies to detect early signs of fetal anemia, which may indicate rupture of the aneurysm or thrombosis. (2) Non-stress testing twice weekly as part of assessment of fetal wellbeing. (3) Multidisciplinary team approach with relevant stakeholders including neonatology team so that early planning and co-ordination may improve neonatal outcomes. (4) Timing of delivery as determined by clinical parameters; with reasonable consideration at 32 to 36 weeks gestation if the aneurysm remains stable in size and no evidence of fetal compromise and (5) consider delivery via cesarean section if the aneurysmal diameter is greater than 5 cm due to increased risk of rupture. While there are no specific pathognomic or characteristic features on cardiotocography related to an umbilical artery aneurysm; identifying any abnormal fetal heart rate patterns in these fetuses would certainly raise concern for the obstetric team to implement the necessary management options accordingly.

## Conclusions

Umbilical artery aneurysm is a rare structural anomaly of the umbilical cord. Recommendations for adequate interval of fetal monitoring and surveillance require further studies with more reported cases to inform prudent management guidelines. When monitoring the aneurysms with ultrasound; change in size as well as Doppler indices play a pivotal role in helping to determine time and mode of delivery and thus allow for a favorable outcome as was seen in this case.
